# Co‐transformation using T‐DNA genes from *Agrobacterium* strain 82.139 enhances regeneration of transgenic shoots in *Populus*

**DOI:** 10.1111/pbi.70159

**Published:** 2025-06-16

**Authors:** Greg S. Goralogia, Cathleen Ma, David S. Taylor, Abigail Lawrence, Victoria Conrad, Ekaterina Peremyslova, Steven H. Strauss

**Affiliations:** ^1^ Department of Forest Ecosystems and Society Oregon State University Corvallis Oregon USA

**Keywords:** *Agrobacterium*, genetic transformation, *Populus*, regeneration, transgenic

## Abstract

Barriers to transformation and regeneration continue to hamper the application of recombinant DNA‐based biotechnologies in most crops, including for gene editing. To tackle this problem, there has been increasing interest in morphogenic regulator genes, which aid in regeneration and are often plant developmental master regulator genes. Using a set of six genes from the T‐DNA of a ‘shooty’ *Agrobacterium tumefaciens* strain first discovered by researchers at INRA (France) in the 1990s, we developed a co‐transformation (‘altruistic’) system where these genes promote the recovery and rate of regeneration of transgenic poplars without the use of exogenous plant growth regulators. This method was more efficient (2.3x) at regenerating transgenic shoots in poplar and reduced the culturing time by approximately 6 weeks relative to the conventional approach. Resulting transgenic shoots were positive for GFP and antibiotic resistance genes but did not integrate the altruistic morphogenic genes from the second strain and were phenotypically normal. Deletion testing revealed that the hormone biosynthesis genes alone were insufficient to induce altruistic shoot production in poplar. Further mutational analysis of each gene identified *6b,* in combination with *iaaH*, *iaaM* and *ipt,* as the major factor required for non‐cell autonomous shoot proliferation. Altogether, our approach highlights the utility of leveraging *Agrobacterium* genes for transformation, especially through co‐transformation, to avoid retaining morphogenic genes in the genomes of clonally propagated plants.

## Introduction

Genetic engineering and gene editing are powerful tools for the manipulation of agronomic traits (Cardi *et al*., 2023). Current gene editing methods in plants most frequently use conventional transgenesis to insert CRISPR/Cas components into the genome, but it is difficult or impossible in the vast majority of species or genotypes outside of model systems (Atkins and Voytas, 2020; Chen *et al*., 2019). Typically, the development of transformation methods involves the customization of plant growth regulators (PGRs) and other media components to optimize *in vitro* culture and regeneration conditions (Bélanger *et al*., 2024). These variables must be balanced alongside an understanding of DNA delivery efficiency (whether by *Agrobacterium* or another agent) and optimal levels of selection agents to limit non‐transgenic escapes but not suppress the regeneration of transformants. Other variables such as explant type, regeneration pathway, preculture conditions and wounding methods can also play important roles. Integrating all of these factors, often with respect to individual genotypes, requires extensive experimentation and often fails to identify successful methods.

The use of morphogenic regulator genes in transformation represents a paradigm shift from conventional tissue culture optimization, whereby genes included in the delivered DNA spur the regeneration of transformed cells (Gordon‐Kamm *et al*., 2019). Depending on the gene's function, these morphogens can be cell‐autonomous or non‐autonomous in their effects and can improve regeneration in many cases in the absence of exogenously supplied PGRs. In monocots in particular, combinations of morphogenic regulator genes have resulted in greatly improved regeneration rates and are often effective in multiple genotypes. Useful genes include the combination of *WUSCHEL (WUS)* and *BABY BOOM (BBM)*, *GROWTH REGULATORY FACTOR 4 (GRF4)* and *GRF INTERACTING FACTOR 1 (GIF1)*, and *WUSCHEL‐RELATED HOMEOBOX 5 (WOX5)*, among others (Debernardi *et al*., 2020; Lowe *et al*., 2016; Wang *et al*., 2022). This has enabled the expansion of transformable genotypes and species, now reaching many genera of the Poaceae (Wang *et al*., 2023). In addition, morphogenic regulators have enabled the use of expanded explant input types, such as juvenile leaf tissue, which removes the need for immature embryo isolation in maize and other grasses (Wang *et al*., 2023).

In dicots, on the other hand, there are fewer reports of successful use of morphogenic regulator genes in transformation. Woody plants are especially challenging, and genes such as *WUS* and *GRF‐GIF1* inhibit transformation and regeneration in *Eucalyptus* and some poplar genotypes, especially when constitutively expressed (Ryan, 2022). Notable exceptions, at least in specific genotypes, include *WUS* in combination with the cytokinin‐producing *ipt* gene from *Agrobacterium, SHOOT MERISTEMLESS (STM), LEAFY COTYLEDON 2 (LEC2)* and *PLETHORA 5 (PLT5)* (Fister *et al*., 2018; Lian *et al*., 2022; Maher *et al*., 2020). *WUS* and *WOX11* have also been shown to improve regeneration in stable transgenic events of a single poplar genotype *in vitro* (Pan *et al*., 2022). Recently, the application of inducible CRISPR activation of genes such as *GRF‐GIF* and *PLT1* can improve regeneration of strawberry and alfalfa, respectively (Zhang *et al*., 2024). Application of some of these genes, including *WOX5* in conjunction with hairy root transformation, can improve shoot regeneration from transgenic root tissue in selected genotypes of apple and kiwifruit (Liu *et al*., 2024).

*Agrobacterium*‐derived genes have also been used to enhance the regeneration of transformed cells. The cytokinin‐producing *ipt* gene, reported nearly 30 years ago, continues to see use for that purpose (Ebinuma *et al*., 1997; Endo *et al*., 2002b; Maher *et al*., 2020). Hairy root‐inducing genes have seen widespread use in otherwise‐recalcitrant species (Goralogia *et al*., 2025), mostly still encoded on the wild strain T‐DNA, but occasionally have been cloned out of their native context (Ebinuma and Komamine, 2001). In addition, strains have previously been characterized which naturally induce shoots from ‘shooty’ galls. Examples include the 82.139 strain isolated from a wild cherry specimen near INRA (Orleans, France by Dr. Lise Jouanin) and colleagues during the 1990s (Brasileiro *et al*., 1991). Another notable example is the AKE10 ‘shooty’ strain isolated in Japan from an apple specimen (Wabiko and Minemura, 1996) and the T37 nopaline‐type strain (Binns, 1983). The 82.139 and AKE10 strains were studied for their utility in transformation or *in vitro* regeneration in multiple species, including poplar, wild cherry, *Eucalyptus* species and hybrids, silver birch, tobacco, Asian pea pear, *Digitalis minor* and English Elm (Aronen *et al*., 2002; Azmi *et al*., 1997; Brasileiro *et al*., 1991; Fenning *et al*., 1996; Kaneyoshi *et al*., 2001; Sales *et al*., 2002; Wabiko and Minemura, 1996). However, due to the limitations of cloning technology of the time, the sets of causative genes received limited study and had not been employed as direct agents to promote regeneration (i.e. they remained within the wild strains or still possessed opine synthesis machinery; Drevet *et al*., 1994; Kaneyoshi *et al*., 2001).

For clonally propagated crops, many of which are woody dicot species, the use of morphogenic regulators can be problematic, as crossing away the genes will violate the clonal integrity of the cultivar (Goralogia *et al*., 2021). Thus, the use of non‐integrating (i.e. transient) or gene excision approaches is ideal for these species to avoid pleiotropic effects in transgenic products because of morphogenic gene expression. Though excision strategies using, for instance, Cre recombinase are common for *WUS/BBM* systems in maize and other monocots, controlling the expression of the recombinase during regeneration can take considerable optimization, and silencing/toxic effects of Cre expression in dicots have been frequently reported (Coppoolse *et al*., 2003; Liu *et al*., 2021; Wang *et al*., 2023). Another option to utilize morphogenic regulators is to use a so‐called ‘altruistic’ delivery approach which relies on co‐transformation of two T‐DNAs (Gordon‐Kamm *et al*., 2019; Hoerster *et al*., 2020). In this configuration, an *Agrobacterium* strain carrying the morphogen‐encoded T‐DNA is mixed with a second strain containing a ‘trait’ T‐DNA of interest. If the morphogen is sufficiently non‐cell autonomous in its mechanism of action, it can spur the regeneration of surrounding cells to which only the trait T‐DNA was delivered. The benefit of this approach for clonal plants is that transgenic shoots can be recovered which lack the morphogenic gene T‐DNA, thus avoiding pleiotropic effects in the products.

In this study, we explored whether genes derived from strain 82.139 can be leveraged as morphogens to improve transformation in woody dicots, using an altruistic approach and poplar as a testing platform. We report improved transformation rates and speed of transgenic plant recovery in two genotypes with these genes compared to conventional methods. In addition, through mutational studies, we identify the *6b* gene as a major non‐cell autonomous factor for shoot recovery.

## Results

### A set of six genes from 82.139 induce non‐transgenic shoot formation

**Figure 1:**
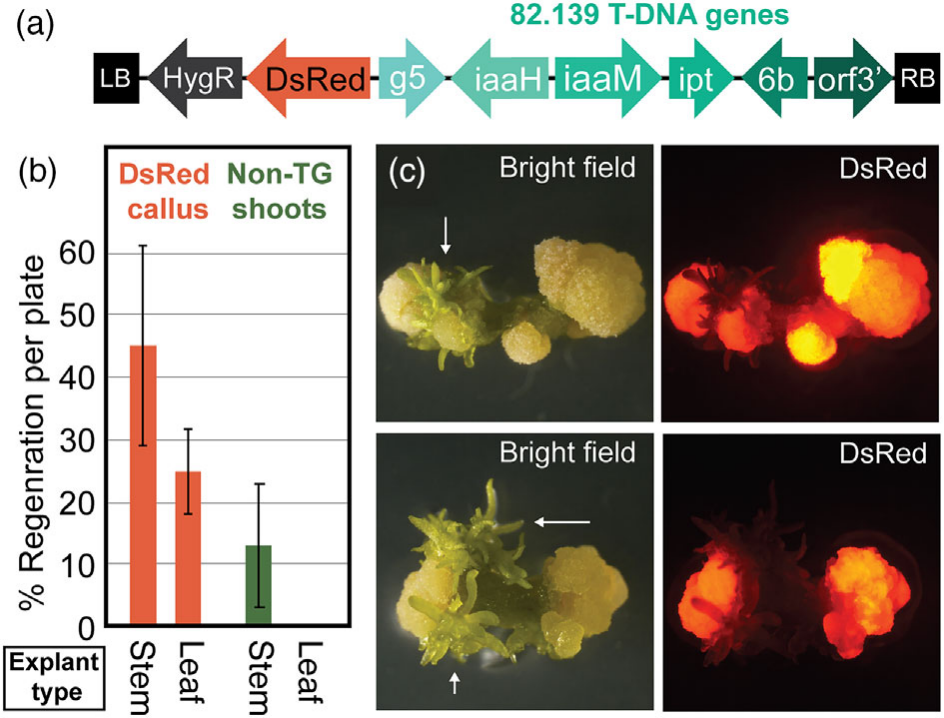
82.139 ‘shooty’ genes encourage the development of non‐transgenic shoots outside of transformed callus tissue. (a) T‐DNA used for study. 82.139 (six‐gene 10kbp region) were delivered by GA*A*NTRY strain ARport1. (b) Percent of 717‐1B4 explants per plate with DsRed callus or non‐transgenic (non‐TG) shoots. No transgenic (DsRed) shoots were observed. (c) Microscopic images of transformants, with white arrows pointing to newly formed non‐transgenic shoot primordia. For (b) and (c), explants were placed on hormone‐free hygromycin‐containing media for 1 month prior to transfer to selection‐free media.

Given the morphogenic qualities reported for strain 82.139, we wanted to understand whether the T‐DNA genes could be useful in improving woody plant transformation and regeneration. To study this, we cloned a six‐gene 10 kilobase pair (kbp) region from the 82.139 T‐DNA, the template of which was embedded in a binary vector‐carrying C58 derivative sent to Oregon State University by Lise Jouanin in 1993. This 10kbp region was placed in a construct where the T‐DNA region is assembled on the *vir* plasmid of strain ARport1 (GA*A*NTRY‐compatible, not on a binary plasmid), alongside a DsRed gene and a hygromycin resistance gene near the T‐DNA left border (Figure 1a) (Collier *et al*., 2018). We transformed this construct into poplar leaf and stem explants of clone 717, then placed them on hygromycin selection for 1 month. We observed high rates of transgenic callus production from leaf and stem explants, but no transgenic shoot production on hormone‐free medium. Surprisingly, we did observe the frequent formation of non‐transgenic escape shoots on the periphery of transgenic callus tissue in stem explants (Figure 1b,c). After removal of the explants from selection, the shoots continued to develop and elongate.

### Altruistic transformation using 82.139 genes

**Figure 2:**
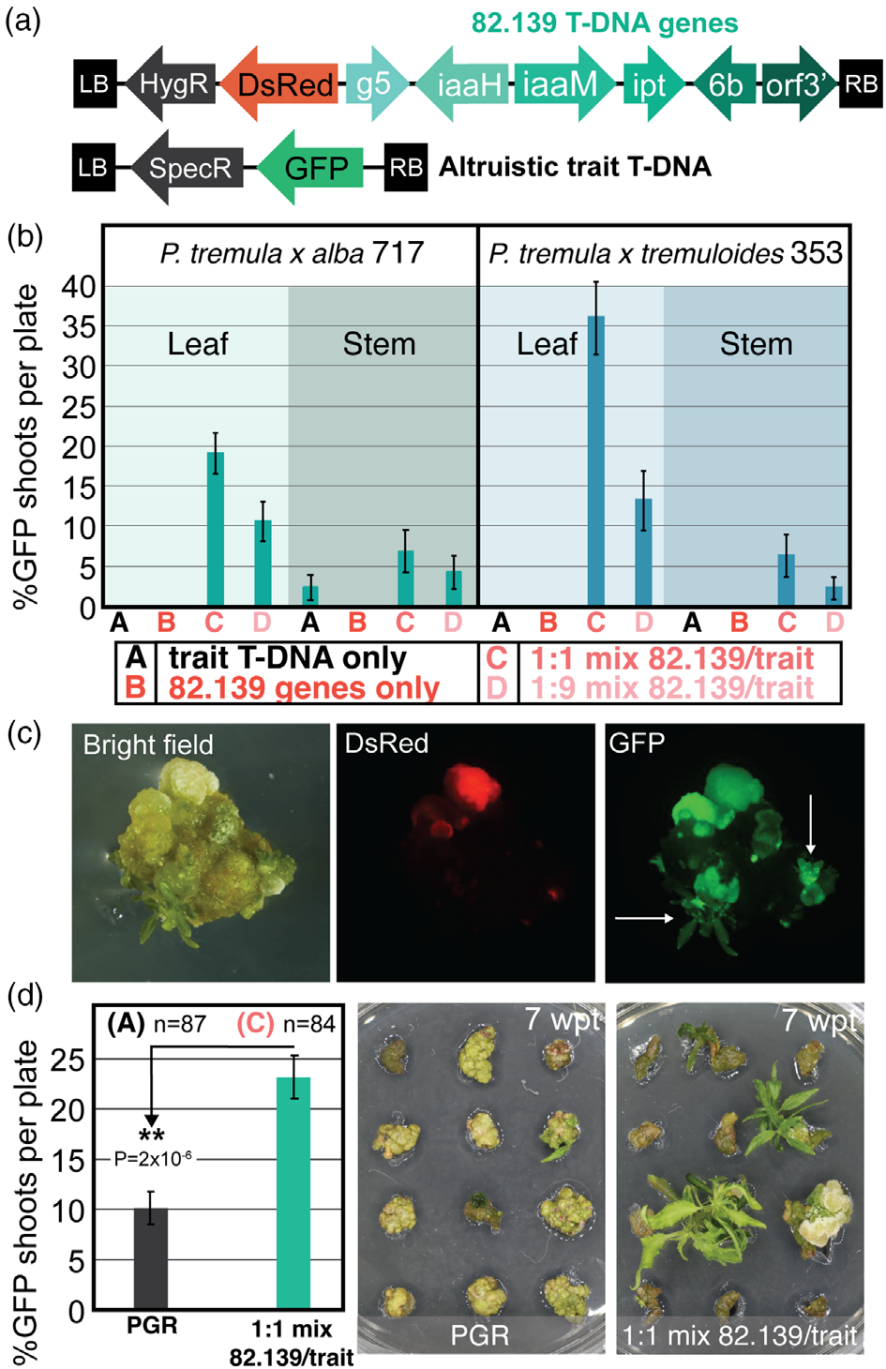
‘Altruistic’ co‐transformation of 82.139 genes enables more efficient and faster transgenic recovery in two poplar clones than a conventional plant growth regulator (PGR)‐based approach. (a) T‐DNAs used for study. 82.139 genes were delivered by GA*A*NTRY strain ARport1, and trait T‐DNA was delivered by LBA4404 (thy‐) in varying ratios or singly. (b) Percent of explants per plate with trait‐T‐DNA‐only transgenic shoots (GFP), in single or mixed constructs with 1:1 or 1:9 ratios, in two poplar clones using leaf or stem explant inputs. No DsRed‐only shoots were recovered in 82.139‐only treatment. Plate inputs per tissue/treatment ranged from four to fourteen with a mean of nine plates per tissue/treatment combination, all with 12 explants per plate. (c) Microscopic images of altruistic transformants, with white arrows pointing to newly formed shoot primordia. Explant shown is from 1:1 strain ratio treatment, 4 weeks after co‐culture. (d) Percent of transgenic shoots containing only GFP (trait T‐DNA) expression using a conventional PGR‐based indirect regeneration method vs. 82.139 altruistic transformation. 4 explants with DsRed shoots were recovered in total (0.05% explants/plate) in the altruistic treatment. *n* = number of plates of 12 explants per treatment. Right panels show transformed shoot elongation at weeks posttransformation (wpt) with each method. *P*‐values shown are pairwise two‐tailed Student's *t*‐tests. ** symbols denote *P* < 0.01.

With the apparent non‐cell autonomous shoot‐inducing signals from the 82.139 genes, we sought to test whether altruistic approaches could be leveraged to produce transgenic shoots which do not integrate the shooty T‐DNA (Gordon‐Kamm *et al*., 2019). We use the term ‘altruistic’ to describe experiments where two constructs are used in a co‐transformation framework; one construct promotes transformation and/or regeneration but does not integrate in the final transgenic or gene edited plant, and one that encodes a ‘trait’ in a transgene intended to remain in, or edit, the derived plant. To test this, we used the previously produced 82.139 GA*A*NTRY strain and mixed immediately prior to explant exposure with another *Agrobacterium* laboratory strain (LBA4404 thy‐) harbouring the trait T‐DNA in a binary vector containing an eGFP gene and a spectinomycin resistance gene (Figure 2a). We transformed two amenable hybrid poplar genotypes, 717 and 353, using single strains (morphogen T‐DNA only, trait T‐DNA only) or strains mixed at 1:1 or 1:9 ratios (morphogen:trait), applying spectinomycin selection after a 1‐week resting phase. In both genotypes, transgenic shoots visibly containing only the trait transgene were produced. Leaf explant input material gave the highest transformation rate in both genotypes (717, 19%; 353, 36%) when 82.139 genes were mixed 1:1 (Figure 2b); 1:9 mixes showed lower trait T‐DNA transformation efficiencies in both genotypes. When viewed by fluorescent microscopy, transgenic shoots containing only trait transgene GFP signal could be seen developing near the periphery of cells expressing DsRed (82.139) or co‐transformed callus expressing both transgenes (Figure 2c). Phenotypically normal shoots were regenerated, and PCR analysis of 14 independent events confirmed the absence of 82.139 genes in those shoots (Figure S1).

To compare the efficiency of 82.139 altruistic transformation *in vitro*, we directly compared 1:1 (morphogen:trait T‐DNA strain) transformation in 717 poplar against our conventional indirect organogenesis protocol with PGRs using only the trait T‐DNA. The 82.139 altruistic method was 2.3x more efficient at producing shoots with only the trait transgene and was significantly faster at producing propagatable shoots (Figure 2d). The size of shoots produced by week seven after transformation shortened the overall production phase by 6 weeks relative to the conventional method and were ready for placement into propagation boxes without the need for a specific shoot elongation medium.

**Figure 3:**
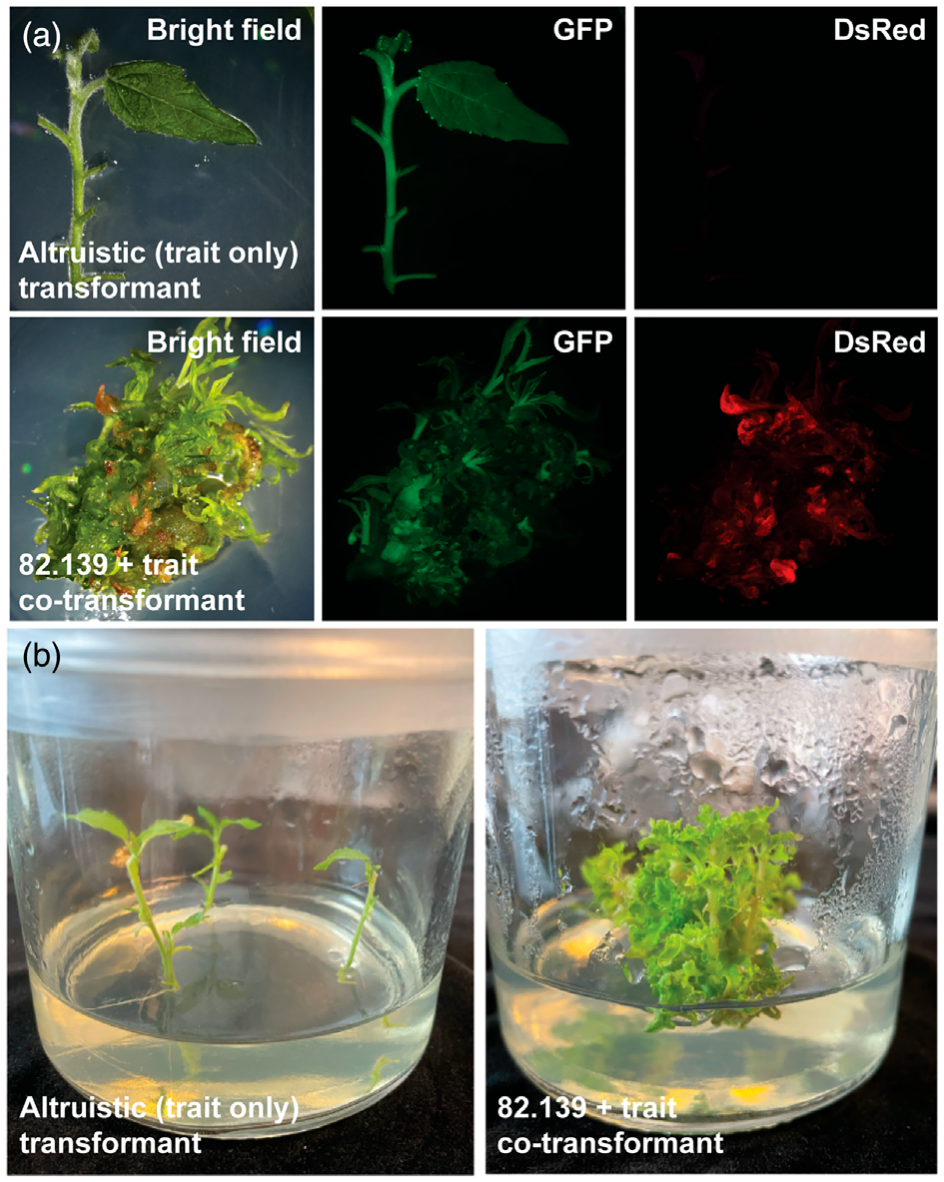
Altruistic transformants that lack 82.139 T‐DNA genes appear phenotypically normal. (a) 717 altruistic trait T‐DNA‐only (upper row) and co‐transformed events (lower row) propagated *in vitro*. (b) Phenotypes of altruistic and co‐transformants after 1 year of *in vitro* propagation with three subcultures. Photographs were taken 3 weeks after last subculture.

To observe any abnormal effects of regenerated altruistic shoots, we transferred spectinomycin‐resistant shoots into magenta boxes and allowed them to grow through several repeated subcultures. Trait‐transgene‐only events had no observable morphological effects *in vitro* and continued to express the GFP marker throughout all tissues (Figure 3a). Shoots where both the morphogen and trait transgenes integrated were morphologically identifiable. These phenotypes included ectopic shoot formation, curled leaves, and severely inhibited rooting (Figure 3b).

### 82.139 Hormone‐producing genes are insufficient to produce altruistic shoots

**Figure 4:**
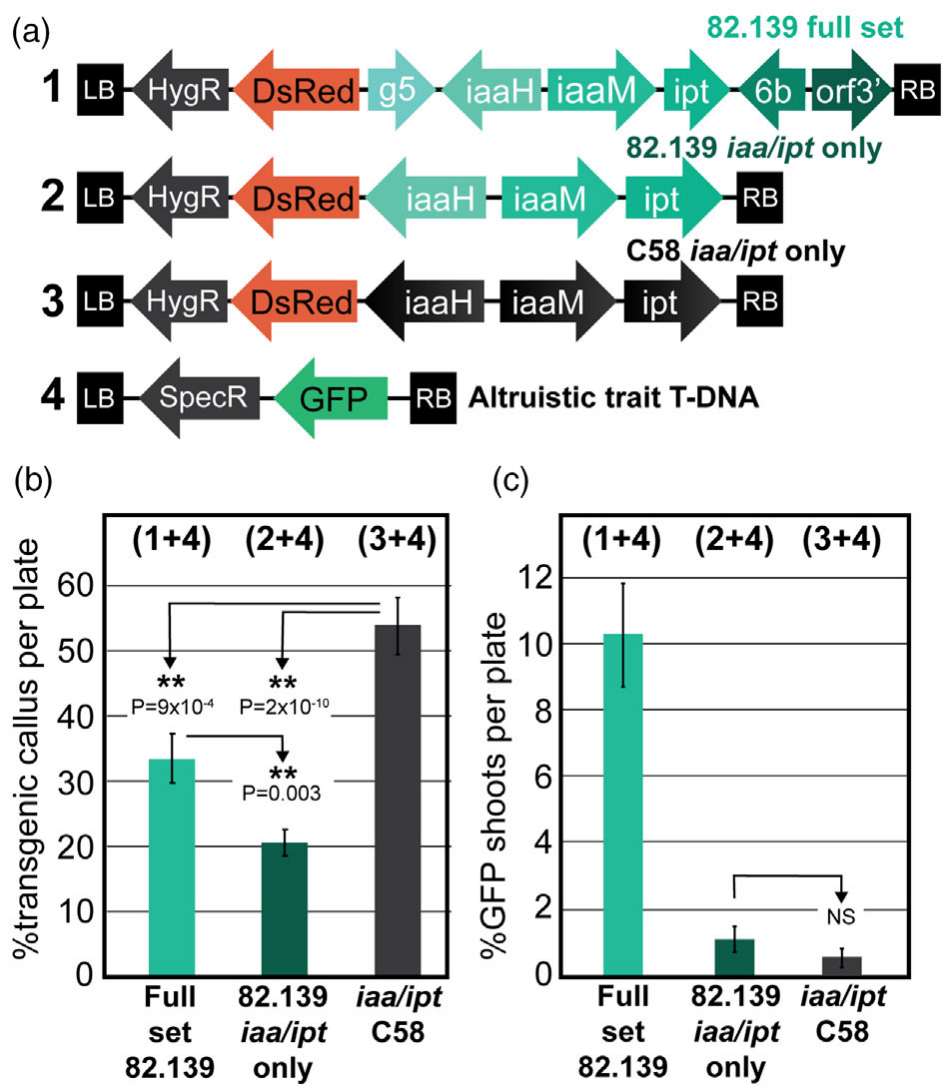
*iaa/ipt* genes from 82.139 are insufficient for altruistic shoot production. All experiments were performed in *P. tremula* × *alba* clone 717‐1B4. Input plate numbers ranged from 45 to 54 per treatment (12 explants per plate) with approximately 600 explants per treatment. (a) T‐DNAs used for study of *iaa* and *ipt* genes isolated from 82.139 or C58. T‐DNAs 1, 2 and 3 were delivered by GA*A*NTRY strain ARport1, and T‐DNA 4 was delivered by LBA4404 (*thy‐*) in 1:1 ratios. (b) Percent of explants per plate with transgenic GFP and/or DsRed callus production with various T‐DNA/trait T‐DNA combinations. (c) Percent of transgenic shoots containing only GFP (trait‐T‐DNA) expression with various T‐DNA/trait T‐DNA combinations. 3 explants with DsRed shoots were recovered in total (0.05% explants/plate) in the 82.139 full set treatment, and none in *iaa/ipt*‐only treatments. *P*‐values shown are pairwise two‐tailed Student's *t*‐tests. ** symbols denote *P* < 0.01.

Because *iaaH*, *iaaM* and *ipt* (hereafter abbreviated as *‘iaa/ipt’*) are known hormone‐producing genes with established non‐cell autonomous functions, we hypothesized that the 82.139 *iaa/ipt* genes might have different expression or protein activities compared to other *Agrobacterium* strains that were responsible for altruistic shoot regeneration. To test this, we cloned the *iaa/ipt* genes from 82.139 and compared them against the full construct of six genes, and against the *iaa/ipt* genes from strain C58 which are well studied (Figure 4a) (Endo *et al*., 2002a; Zhang *et al*., 2015). These were mixed at a 1:1 ratio with the trait T‐DNA strain and transformed in 717. Both 82.139 six‐gene constructs and *iaa/ipt*‐only construct had reduced transgenic callus formation rates vs. the C58 *iaa/ipt* (Figure 4b). Only the 82.139 six‐gene set could induce the regeneration of trait transgene‐only shoots at high rates (Figure 4c).

### 82.139 Gene *6b* + *iaa/ipt* are the main factors required for altruistic shoot regeneration

**Figure 5:**
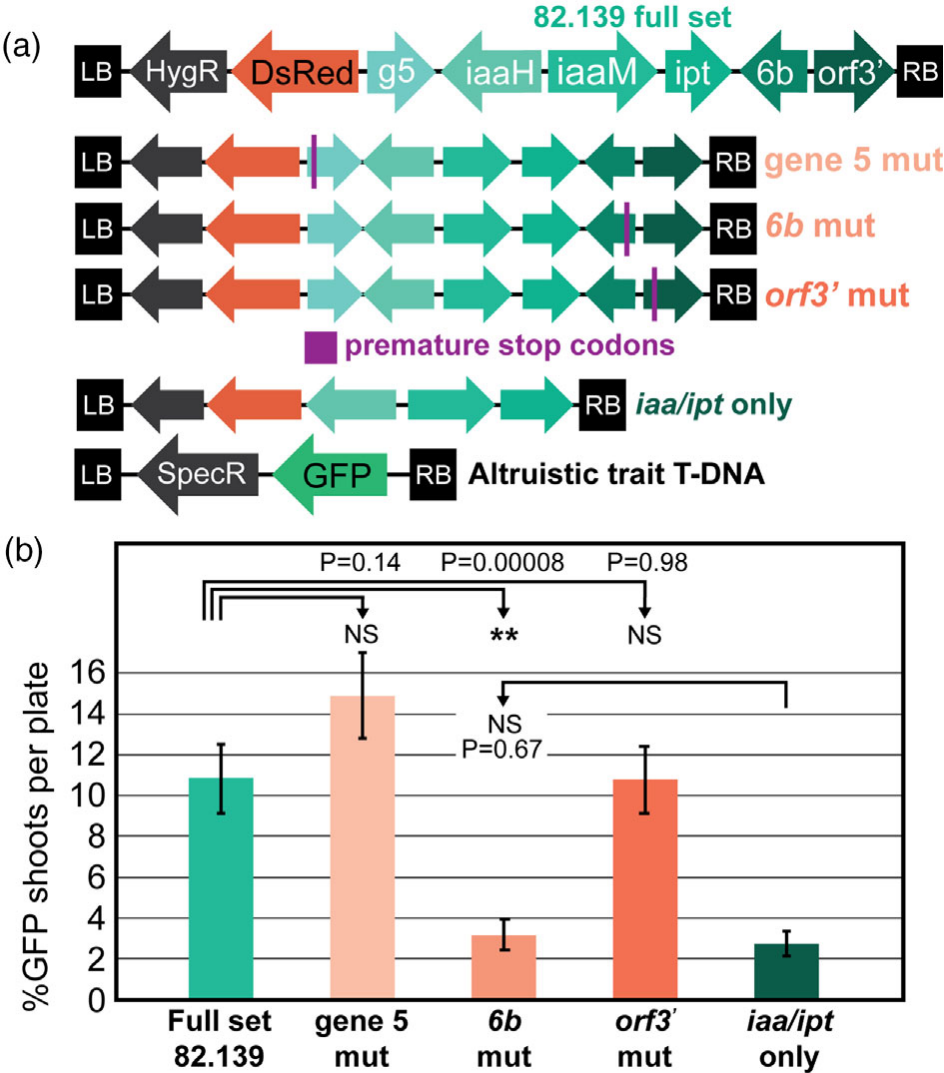
*6b* + *iaa/ipt* genes from 82.139 are required for altruistic shoot production. All experiments were performed in *P. tremula* × *alba* clone 717‐1B4. Input plate numbers ranged from 35 to 50 per treatment, approximately 420–600 explants per treatment in total. (a) T‐DNAs used to study the effect of gene 5, *6b* and *orf3’* on altruistic shoot production. 82.139 genes were delivered by GA*A*NTRY strain ARport1, and trait T‐DNA was delivered by LBA4404 (thy*‐*) in 1:1 ratios. Purple vertical bars indicate introduced premature stop codons. (b) Percent of transgenic shoots containing only GFP (trait‐T‐DNA) expression with various T‐DNA/trait T‐DNA combinations. *P*‐values shown are pairwise two‐tailed Student's *t*‐tests. ** symbols denote *P* < 0.01.

Because the *iaa/ipt* genes alone from strain 82.139 were unable to spur the development of trait transgene‐only shoots, we sought to test the effect of the three other genes in the six‐gene full construct to determine their roles in non‐cell autonomous shoot regeneration. We first reassembled the 82.139 gene cluster into separate plasmid modules so we could generate a deletion series via golden gate assembly with binary vectors. A compromise with this approach was a change to the gene order placing gene *6b* near the T‐DNA left border. When launched from the same strain, the Golden Gate‐assembled version was able to produce trait transgene‐only shoots but was less efficient compared to the previous version launched on the GA*A*NTRY *vir* plasmid (Figure S2, 15 vs. 10%), and had larger callus tissue. To retain the exact properties of the 82.139 T‐DNA from our previous studies, we introduced premature stop codons into the coding sequence of gene 5, gene *6b* and *orf3’* to produce single gene knockouts, and delivered them from the same GA*A*NTRY ARport1 strain. These constructs resulted in predicted 11–18 amino acid products before the introduced stop codons, while only requiring 1 bp changes in the 10kbp region to retain the nearly identical sequence and gene order (Figure 5a).

**Figure 6:**
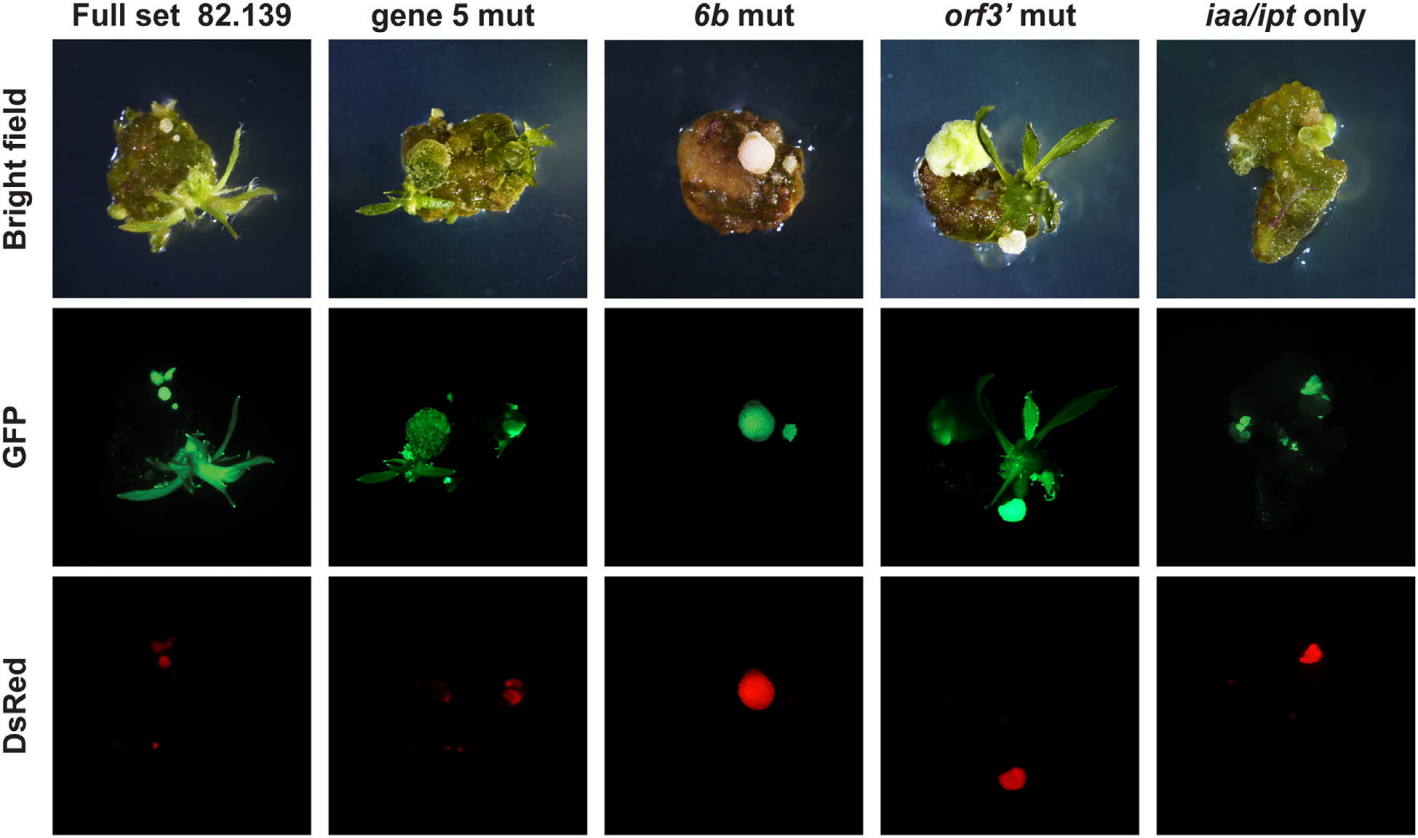
Mutations in the *6b* gene from 82.139 resulted in a loss of non‐cell autonomous shoot‐forming signals. Representative brightfield and fluorescent micrographs of *P. tremula* × *alba* clone 717‐1B4 transformed with 82.139‐related T‐DNAs (see Figure 5). Strains were mixed at 1:1 ratios and imaged at 5 weeks after co‐cultivation.

We transformed each single gene knockout into 717, mixed at 1:1 ratios with trait T‐DNA and compared each against the full six‐gene construct and the *iaa/ipt*‐only construct as a negative control. The single gene knockout of gene 5 had a higher mean altruistic shoot production compared to the full six‐gene construct (14.5% vs. 10.2%, respectively), but were not significantly different (*P* = 0.14, Figure 5b). *orf3'* single knockouts had nearly identical efficiencies compared to the full six‐gene construct and were also not significantly different (*P* = 0.98). Gene *6b* single knockouts were drastically less efficient than full set controls and were comparable to *iaa/ipt‐*only negative controls (2.5% vs. 2.2%, *P* = 0.67, Figure 5b). Resultant trait‐T‐DNA‐transformed shoots were phenotypically normal like prior experiments with the full set construct, and *6b* mutants failed to form shoots at the periphery of the transgenic callus (Figure 6).

To test whether *6b* overexpression alone could induce similar morphogenic outcomes, we compared the full six‐gene construct against a similar T‐DNA delivering *6b* expressed under a constitutive 35S promoter and included *iaa/ipt* alone as a negative control. These were carried out similarly to other experiments without PGRs in the medium. While constructs with the full six‐gene were capable of altruistic trait T‐DNA‐only shoot regeneration, neither the *iaa/ipt* genes nor *35S:6b* alone gave rise to regenerated shoots (Figure S3).

## Discussion

### Co‐transformation of *Agrobacterium* ‘shooty’ genes is efficient for transgenic poplar production

In this study, we showed the utility of *Agrobacterium* genes from a naturally shooty strain to aid the production of transgenic plants in a woody dicot species, poplar. A co‐transformation approach using these cloned genes as an altruistic morphogen is advantageous to produce transgenic clonally propagated crop species as the morphogenic genes do not integrate into the plant genome, preserving clonal integrity (Figure S1). The derived plants are therefore phenotypically normal *in vitro* without the segregation of the T‐DNA in progeny (as would occur in annual plants). Plants with the trait transgene alone were easily distinguishable from ones with morphogenic transgene integration, but further greenhouse trials will be required to determine whether risks of somaclonal variation are increased in the presence of these *Agrobacterium* genes rather than through the application of exogenous phytohormones.

### *6b* in combination with *iaa/ipt* are the factors required for altruistic shoots

The six‐gene shooty T‐DNA region includes several genes that have had variable levels of scientific analysis of their functions. Gene 5, the most upstream, is reported to be an indole‐3‐lactate synthase. Its function is suspected to modulate the plant sensitivity to auxin (Körber *et al*., 1991). IaaH and IaaM are well studied and function as auxin biosynthesis enzymes (Zhang *et al*., 2015). Together they act as two steps in a parallel pathway to catalyse the formation of indole acetic acid (IAA) from tryptophan using indole‐3‐acetamide (IAM) as an intermediate (Mashiguchi *et al*., 2019). Ipt protein is localized to plastids and functions by catalysing the reaction between 1‐hydroxy‐2‐methyl‐2‐(E)‐butenyl 4‐diphosphate (HMBDP) to trans‐Zeatin type cytokinins, bypassing natural cytokinin biosynthesis in plants (Sakakibara *et al*., 2005). *orf3’*, the most distal gene towards the T‐DNA right border before the nopaline synthesis gene, belongs to the phenotypic plasticity (PLAST) family that includes *rolB* and *6b* (Otten, 2018). *orf3’* is poorly studied, though constructs containing a partial fragment of the AKE10 shooty T‐DNA including *orf3’* lost the ability to cause shoot proliferation in tobacco (Wabiko and Minemura, 1996). Although the early hypotheses about the mechanistic nature of the shooty tendency of this strain was differential expression of *iaa* and *ipt* (Drevet *et al*., 1994), our experiments determined that the hormone‐producing genes alone were insufficient for shoot production; similarly, we showed that *orf3’* and *gene 5* were not essential for altruistic shoot induction.

Similar to studies of strain AKE10, we determined that the *6b* gene in 82.139, alongside *iaaH*, *iaaM* and *ipt*, is the primary factor required for the non‐cell autonomous proliferation of shoots (Wabiko and Minemura, 1996). Gene *6b* has several reported functions in the literature. The AKE10 6b is reported to interact with the NtSIP1/2 transcription factors in tobacco and thus putatively functions as a transcriptional activator or repressor (Kitakura *et al*., 2002). *6b* genes present in naturally transformed plants (as a result of horizonal gene transfer during their evolution) similarly function to change the localization patterns of TCP4 transcription factors, resulting in changes to development (Potuschak *et al*., 2020). 6b proteins also interact with different histone subunits, including histone H3, and thus may also function in some chromatin‐dependent regulation (Terakura *et al*., 2007). AKE10 6b has also been shown to bind to both ARGONAUTE 1 (AGO1) and SERRATE (SE) to function in downregulating miRNA biogenesis in a nonspecific manner (Wang *et al*., 2011). This occurs via innate ADP‐ribosylation activity in combination with the Arabidopsis factor AtARF1 to negatively impact the function of AGO1 and SE. In addition to these functions in manipulating plant development and gene expression, 6b also binds to VFP3/5 protein (Arabidopsis NtSIP1 homologues) involved in secreted VirF signalling and may modulate the host defence response during early *Agrobacterium* infection (García‐Cano *et al*., 2015). Given the wide array of potential mechanisms of how 82.139 *6b* acts, it is presently impossible to specify how it provides a non‐cell autonomous shoot‐inducing signal required for altruistic transformation. Although 6b protein or mRNA might be cell‐to‐cell mobile, given the known mechanisms of 6b activity (including interference with miRNA biogenesis and protein sequestration of TCP transcription factors), another hypothesis is that these activities within gall/callus tissues lead to the export of factors, such as hormones, to neighbouring tissues that result in stimulation of shoot regeneration.

To better understand differences in 82.139T‐DNA genes relative to T‐DNA genes encoded on Ti plasmids of other strains, we aligned the *6b* protein sequence against those selected from Ti clusters defined in Weisberg *et al*. (2020); *6b* genes are common among Ti clades (Weisberg *et al*., 2020). The 82.139 6b protein has three motifs that are similar in sequence to 6b proteins from type IVa Ti plasmids and distinct from most other Ti lineages (Figure S4a). These regions were previously characterized from AKE10 with exactly the same amino acid sequence as 82.139 6b, as the target‐binding loop near the N‐terminus and the active‐site loop in the middle of the peptide (Wang *et al*., 2011). In addition, there is a region near the C‐terminus with many repeated glutamic acid residues that is extended in 82.139 relative to those in other clades (Figure S4a).

Despite the mechanistic uncertainties, our approach has shed some light on the action of the 82.139 genes in regeneration. Firstly, our study of the T‐DNAs has revealed that the production of shoots is at the distal sections of transgenic callus and explains why some tissues obtained in earlier studies, such as in silver birch, were highly chimeric (Aronen *et al*., 2002). Tuning of selection agents, timing and concentration appears critical to use these genes as transformation tools. In addition, our sequence analysis from newly available genome sequences suggests the recombined region including *6b* in type 1a Ti plasmids comes from type IVa *A. vitis* strains, and not from type II plasmids like Ach5, as was suggested historically for 82.139 (Drevet *et al*., 1994; Weisberg *et al*., 2020).

### Factors for translating to other dicot species

In developing this gene cluster as a transformation tool in poplar, several factors emerged that may be important for translating this system into other dicot crop species. Firstly, the ratios of the morphogenic strain to the trait strain are critical. Excess morphogenic T‐DNA may result in abundant co‐transformed cells trapped in a non‐regenerable gall developmental fate, while too little and the trait‐T‐DNA‐transformed cells will not regenerate due to insufficient morphogen. The rate of transformation and distribution of transformed cells may change based on the strain from which each T‐DNA is launched, as well as whether it is launched from a binary, *vir* plasmid, or from a T‐DNA encoded on the *Agrobacterium* chromosome (the latter is a useful option to reduce 82.139T‐DNA copy number; Oltmanns *et al*., 2010; Collier *et al*., 2018). Testing different ratios and possibly different explant sizes in new species would be important starting points when developing a protocol. Selection agent choice and concentration are also critical. In this study, the use of spectinomycin as a selection agent lessens the negative impact on 82.139‐only transformed callus, as spectinomycin primarily impacts shoot tissue (Cho *et al*., 2022). A resting phase of 1 week prior to the start of selection, as we carried out, likely helped trait‐T‐DNA‐transformed cells to develop (Hoerster *et al*., 2020). When using other antibiotics, care must be taken to balance positive trait T‐DNA selection against the negative impacts of dying, potentially necrotic 82.139‐only transformed cells.

### Many *Agrobacterium* genes remain unexplored as tools for plant transformation

Our study highlights that despite extensive work in the 1980s–1990s to understand the function of genes encoded on the *Agrobacterium* Ti or Ri plasmid T‐DNAs, there are significant gaps in the mechanistic underpinnings of these genes, or whether they might have utility in improving plant transformation. For example, given the wide variation in *iaa* and *ipt* AuxRE and ARR motifs among strains (Figure S4b), it would be of interest to test whether different *iaa/ipt* gene sets of independent strain origins, in combination with the 82.139 *6b* gene, might provide a ‘palette’ of different auxin/cytokinin ratios upon which 6b or other morphogenic proteins can act. Such construct suites might provide tools to help adapt altruistic transformation systems to other species, mirroring how humans manipulate these ratios in tissue culture with PGRs. However, as these are expressed only from transformed tissues, they may provide important further tools to refine both PGR‐ and morphogen‐mediated plant transformation.

With modern cloning and editing techniques, the combination and engineering of genes from various *Agrobacterium* strains that do not exist together in the wild might be a promising approach to produce transformation tools tailored to different species. In addition to well‐studied genes such as *iaaH/M*, *ipt* and *rolB*, recent sequencing surveys of hundreds of Ti and Ri plasmids reveal many T‐DNA‐encoded genes of unknown function, some of which could impact plant development in ways that could be leveraged for plant transformation (Otten, 2021; Weisberg *et al*., 2020). Synthetic circuits using the known and novel genes, possibly in combination with morphogens derived from plants, might be able to exert beneficial responses to produce transgenic or edited lines in hitherto recalcitrant species. The non‐cell autonomous nature of many *Agrobacterium* T‐DNA genes makes them promising candidates for altruistic modes of transformation, which would be of particular value in clonally propagated crops for which sexual removal of morphogenic genes is undesirable.

## Experimental procedures

### Construct assembly

T‐DNAs containing *Agrobacterium* genes as morphogens were constructed using GA*A*NTRY strain ARport1 (Collier *et al*., 2018). B donor and P donor plasmids for iterative stacking were modified to accept MODA, MODB and MODC input plasmids for Golden Gate multigene assembly derived from (Čermák *et al*., 2017), using the *Paq*C1‐flanked region containing ccdB and chloramphenicol resistance genes from pTRANS200, flanked and inserted into *Hind*III and *Sac*I sites. 82.139 six‐gene sets encompassing a 10kbp region were cloned into P donor plasmids in two stages. A large fragment including the region from *iaaH* to *orf3'* was amplified using Q5 polymerase (New England Biolabs), containing restriction enzyme overhangs with *Sbf*I and *Asc*I. A second smaller fragment containing gene 5 was amplified and used a natural *Xba*I upstream site and corresponding *Sbf*I site to produce the previously studied T‐DNA without the nopaline encoding region (Drevet *et al*., 1994). The template used to produce the region was the 82.139 T‐DNA *Xba*I fragment in a binary vector of unknown origin, placed in a disarmed C58 strain that was shipped to Oregon State University in 1993 (Drevet *et al*., 1994; Koncz and Schell, 1986). Hormone‐only gene sets from C58 and 82.139 were amplified in a similar manner and ligated into corresponding P donor plasmids in the same orientation as the six‐gene 82.139 constructs. Final GA*A*NTRY assembly (two stacks) followed the methods outlined in (Collier *et al*., 2018), but differed in that assembly was accomplished using room‐temperature electroporation (Tu *et al*., 2016), and confirmation of the loss of the *sacB* gene was done in liquid culture using 150 mM sucrose.

Trait T‐DNAs were produced using the same (Čermák *et al*., 2017)‐derived input plasmids but mobilized in binary vector pTRANS200. The p35S:eGFP‐35Sterm‐minRb7MAR gene was built based on Diamos and Mason, 2018 and was previously published in Nagle *et al*., 2024. The spectinomycin resistance gene was derived from Cho *et al*., 2022, generously provided by Corteva Agriscience. Trait T‐DNAs were mobilized in a thymidine auxotrophic mutant of *Agrobacterium* strain LBA4404 (thy‐), also generously provided by Corteva Agriscience (Hoerster *et al*., 2020).

Single gene mutations of non‐hormone encoding factors were produced via site‐directed mutagenesis. Mutagenic primers were used that contained premature stop codons, where mutagenic primer pairs were used to generate hybrid fragments for overlap‐extension PCR to reassemble a larger region using internal restriction enzyme sites. Nested PCR was used for final fragment production (long‐PCR fusion) prior to restriction enzyme digest and ligation (Shevchuk *et al*., 2004). Sequences of the assembled T‐DNAs are given in Data S2. Primers used to amplify T‐DNA genes or perform site‐directed mutagenesis are given in Data S1.

### Plant materials

In this study, two diploid hybrid poplar genotypes were employed, *Populus tremula* × *alba* 717‐1B4 (hereafter abbreviated ‘717’) and *Populus tremula* × *tremuloides* 353–53 (hereafter abbreviated ‘353’), both a product of research at INRA, France. *In vitro* materials for plant transformation were subcultured in magenta boxes using ½ MS medium supplemented with 2% sucrose, 200 mg/L L‐glutamine, 250 mg/L 2‐(N‐morpholino) ethanesulphonic acid (MES), 100 mg/L myo‐inositol, 10 mL/L F‐vitamin cocktail and 7 g/L agar. Additionally, clone 353 had IBA supplemented to 0.1 mg/L in the medium. Materials from each genotype were freshly subcultured by shoot tips or nodal segments between 40 and 60 days prior to transformation.

### Plant transformation

Media formulations and culture times/conditions are given in Supplemental Table S1. Each *Agrobacterium* strain was grown in liquid overnight culture to saturation in 50 mL YEP in 250 mL flasks using 1 mL of freshly grown inoculum. Cultures were pelleted in a bucket tabletop centrifuge (Beckman) in 50 mL falcon tube format and resuspended in MS‐induction medium at an O.D. = 0.5. Morphogen and trait T‐DNA strains were mixed in specific ratios, then treated with 100 mg/L acetosyringone and incubated for 1 hour prior to co‐culture with explants. Explants were derived from 4 mm leaf discs produced with hole punches, or stem and petiole segments, excluding axillary buds, cut with a scalpel to 3‐4 mm and wounded with multiple fine cuts. All input tissues from multiple magenta boxes were mixed in a single pool, then distributed to each construct mixture to randomize source input tissues exposed to each treatment. Explants were incubated with *Agrobacterium* mixtures for 1 hour of liquid infection on a shaker table at ~60 rpm prior to plating on semisolid CIM + thy medium for co‐culture. Leaf disc materials were placed with the abaxial side down.

After co‐culture, explants were washed in sterile water five times, blotted on sterilized paper towels and plated on CIM0TCR, containing rifampicin, timentin and cefotaxime to eliminate strain ARport1. Explants stayed on this resting medium without plant selection for 1 week and were subsequently transferred onto CIM0TCR + Spec, a hormone‐free medium with selection for the trait T‐DNA alone. Plates were kept in growth chambers (Percival) in the dark at 22C for 3 weeks before being transferred to light conditions with 16/8 h L/D photoperiods at 25C and 40–60uM light. Explants were subcultured on the same media every 3 weeks.

### Statistical analysis

Scoring of transgenic shoots and callus occurred on week six after transformation for all altruistic experiments, and week 10 when shoots were at the same stage of development when using PGRs in the media (conventional transformation). Transgenic shoot regeneration efficiency was accomplished by counting the number of explants per plate which contained at least one transgenic shoot (using GFP fluorescence), divided by the total number of explants per plate (all were standardized to 12 viable explants/plate). Transgenic callus was similarly scored by counting the number of explants which had regenerating callus with GFP, DsRed or both reporter signals. Ranges of plate numbers per construct in each experiment are given in the figure legends. Means, standard deviations (σ) and standard error (σ/√n) within construct combinations or treatments were computed on these percent observations per plate, which were treated as individual replicates for statistical analysis. *P*‐values were determined by two‐tailed Students's *t*‐tests for pairwise comparison of constructs or other treatment conditions. *P*‐values <0.05 are denoted by * symbols, and those <0.01 are denoted by ** symbols. Non‐significant comparisons are denoted by NS.

### Fluorescence microscopy and photography

Fluorescence microscopy was performed on a Nikon SMZ25 stereomicroscope on a motorized chassis, using a TRITC longpass filter for DsRed and a GFP bandpass filter for eGFP to eliminate red chlorophyll emission. Brightfield plate images were taken using a Macrophor Array imager (Middleton Spectralvision) set to take RGB‐only images (Nagle *et al*., 2024). Images of micropropagated plants were taken on an iPhone 12 (Apple).

### Transgene DNA analysis

Testing for integration of trait‐transgene‐only vs. both morphogen and trait transgenes was accomplished by PCR using primers amplifying part of the spectinomycin resistance gene on the trait T‐DNA or primers amplifying the 82.139 *orf3'* coding sequence to detect the morphogenic T‐DNA. DNA was extracted from plant leaves using the Edward's method (Edwards *et al*., 1991), and regions were amplified using Q5 polymerase (New England Biolabs), using a 65 °C annealing temp and 30s extension time, with 35 cycles performed.

### Protein alignment and cis‐element comparison

Sequences for other T‐DNA regions were obtained from (Weisberg *et al*., 2020). *6b* genes and the promoter regions of *iaaH/iaaM* and *ipt* were identified by homology to C58. 6b protein alignments were performed using Clustal‐omega https://www.ebi.ac.uk/jdispatcher/msa/clustalo and plotted using pyBoxshade. https://github.com/mdbaron42/pyBoxshade. AuxRE site numbers were counted using the TGTCNN core site (Mironova *et al*., 2014), and ARR‐type cis‐elements were calculated using the stringent AGATHY (Xie *et al*., 2018) or permissive GATT (Zhang *et al*., 2015) sequences. Promoters were defined by the bidirectional region between *iaaH* and *iaaM* coding sequences and the 500 bp region upstream of the *ipt* or *6b* gene start codon.

## Conflict of interest

The authors report no conflicts of interest regarding this work.

## Author contributions

GSG and SHS designed the study. Transformation experiments were performed by CM and EP. Cloning of constructs was performed by GSG, DST and AL. Microscopy and DNA analysis were performed by GSG, DST, AL and VC. Results were interpreted by GSG, CM and SHS with input from other authors. GSG and SHS wrote the manuscript, and GSG prepared figure illustrations. All authors contributed revisions.

## Supporting information


**Figure S1** Altruistic transformants lack wild T‐DNA genes but integrate trait T‐DNA. Both gels represent 14 independent transgenic events and a wild‐type negative control (15), with two phenotypically abnormal shoots indicative of 82.139T‐DNA integration as shown by arrows and green labels (5,14). (a) Amplification of a ~700 bp fragment encompassing the spectinomycin resistance gene. (b) Amplification of a ~1500 bp fragment encompassing the *orf3'* gene region in the 82.139T‐DNA.

**Figure S2** Golden Gate‐reassembled 82.139 genes with gene order changes are less efficient than the wild fragment. (a) Construct schematic for Golden Gate‐compatible assembly of 82.139 genes. Version 1 and version 2 are delivered by GA*A*NTRY strain ARport1 and the altruistic trait T‐DNA is delivered by LBA4404 (thy‐). Both version 1 and version 2 were mixed 1:1 with the altruistic trait T‐DNA‐carrying strain. (B) Percent of explants per plate with trait‐T‐DNA‐only transgenic shoots (GFP). (C) and (D) show representative plate images of version 1 and version 2 constructs 7 weeks after co‐cultivation. Shoots from version 2 were morphologically smaller, and explants contained larger callus tissue compared with the original version 1 construct.

**Figure S3** Overexpression of 82.139 *6b* is insufficient for altruistic shoot production. All experiments were performed in *P. tremula* × *alba* clone 717‐1B4 using 11–15 plates per construct and 12 explants per plate. (a) T‐DNAs used for study of *iaa*, *ipt* and *6b* genes from 82.139. T‐DNAs 1, 2 and 3 were delivered by GAANTRY strain ARport1, and T‐DNA 4 was delivered by LBA4404 (thy‐) using 1:1 ratios. (b) Percent of explants per plate with transgenic GFP or DsRed callus production with various 82.139/trait T‐DNA combinations. (c) Percent of regenerated transgenic tissue per plate with shoots containing only GFP (trait T‐DNA) expression using various 82.139/trait T‐DNA combinations. (D) Representative brightfield and fluorescent micrographs of explants transformed with 82.139 + trait T‐DNAs. Explants were imaged at 6 weeks after co‐cultivation. *P*‐values shown are pairwise two‐tailed Student's *t*‐tests. ** symbols denote *P* < 0.01.

**Figure S4** Characteristics of *6b* and hormone‐producing genes in the 82.139T‐DNA relative to other strains. (a) Alignment of 6b protein sequences among strains from phylogenetically representative groups of strains with Ti plasmids; Ti type follows Weisberg *et al*. (2020). Regions identified in Wang *et al*. (2011) for the AKE10 strain are shown in orange. (b) Copy numbers of cis‐elements in the promoter regions of *iaaH/M, ipt* and *6b* among the strains shown in (a). Two ARR cytokinin‐responsive elements (stringent‐center, permissive‐right) were analysed.

**Data S1** Sequences of 82.139 T‐DNA.

**Data S2** Primers and sequences used in this study.

**Table S1** Media compositions.

## Data Availability

The data that support the findings of this study are available from the corresponding author upon request or contained in Supplementary Material of this article. All strains and plasmids will be made available upon request.
