# Supplementary figures and images for: Co‐transformation using T‐DNA genes from Agrobacterium strain 82.139 enhances regeneration of transgenic shoots in Populus
Source: Plant Biotechnol J. 2025 Jun 16;23(9):3841–50. doi: 10.1111/pbi.70159 (PMC12392948; doi:10.1111/pbi.70159)

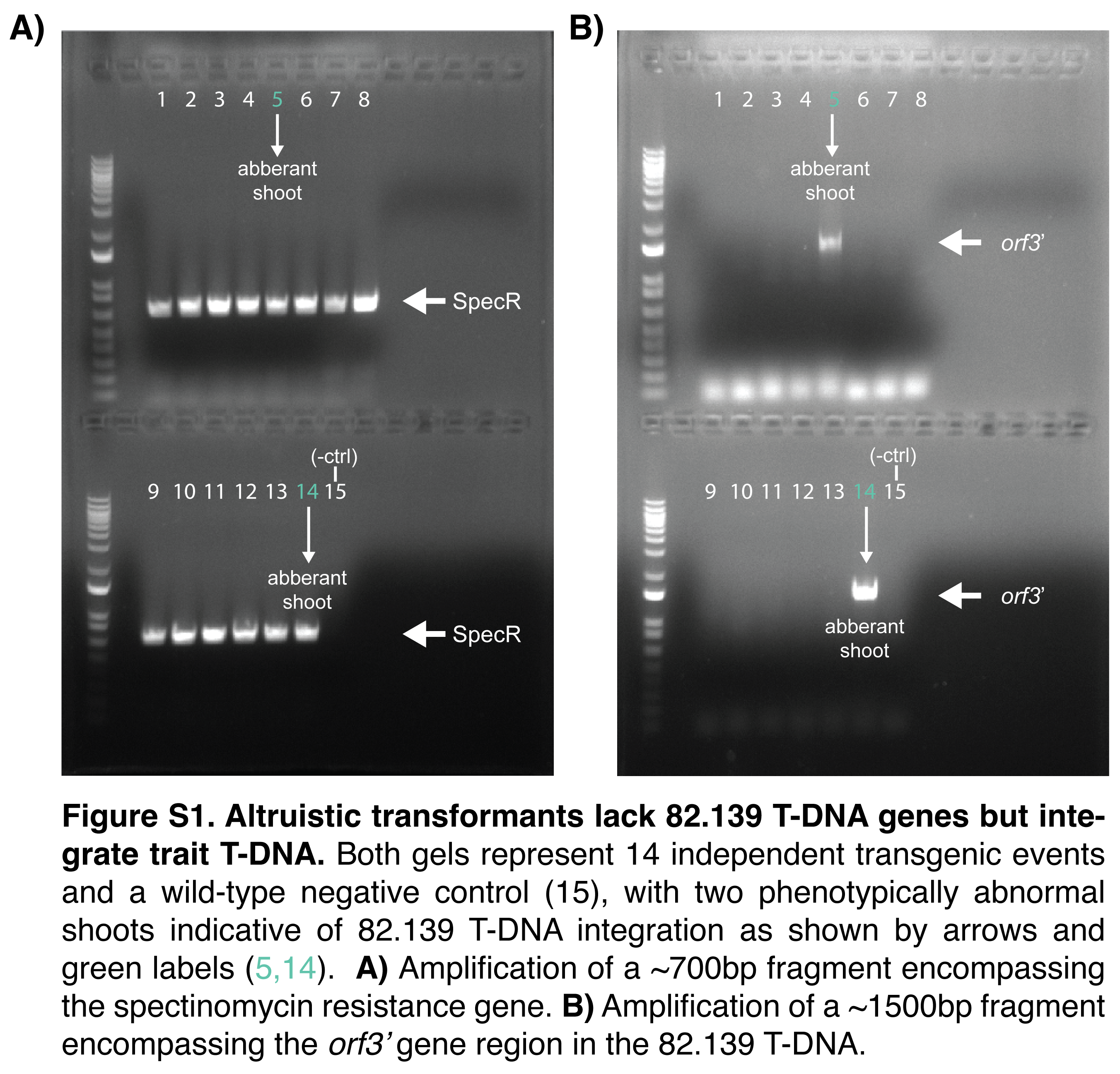

Supplement: Supplementary file 1 — Figure S1 Altruistic transformants lack wild T‐DNA genes but integrate trait T‐DNA. Both gels represent 14 independent transgenic events and a wild‐type negative control (15), with two phenotypically abnormal shoots indicative of 82.139T‐DNA integration as shown by arrows and green labels (5,14). (a) Amplification of a ~700 bp fragment encompassing the spectinomycin resistance gene. (b) Amplification of a ~1500 bp fragment encompassing the orf3' gene region in the 82.139T‐DNA. [file PBI-23-3841-s006.png]

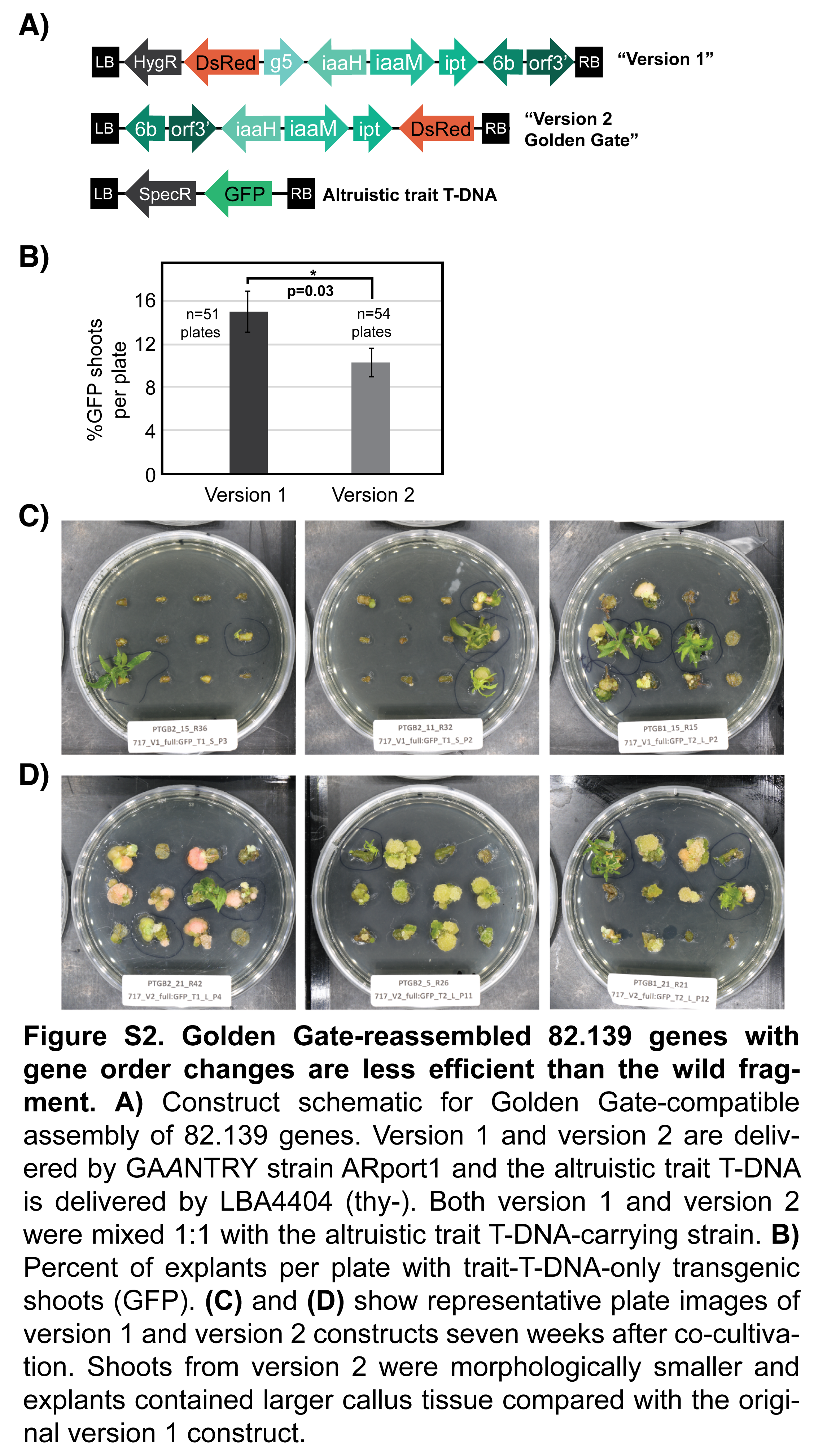

Supplement: Supplementary file 2 — Figure S2 Golden Gate‐reassembled 82.139 genes with gene order changes are less efficient than the wild fragment. (a) Construct schematic for Golden Gate‐compatible assembly of 82.139 genes. Version 1 and version 2 are delivered by GAANTRY strain ARport1 and the altruistic trait T‐DNA is delivered by LBA4404 (thy‐). Both version 1 and version 2 were mixed 1:1 with the altruistic trait T‐DNA‐carrying strain. (B) Percent of explants per plate with trait‐T‐DNA‐only transgenic shoots (GFP). (C) and (D) show representative plate images of version 1 and version 2 constructs 7 weeks after co‐cultivation. Shoots from version 2 were morphologically smaller, and explants contained larger callus tissue compared with the original version 1 construct. [file PBI-23-3841-s007.png]

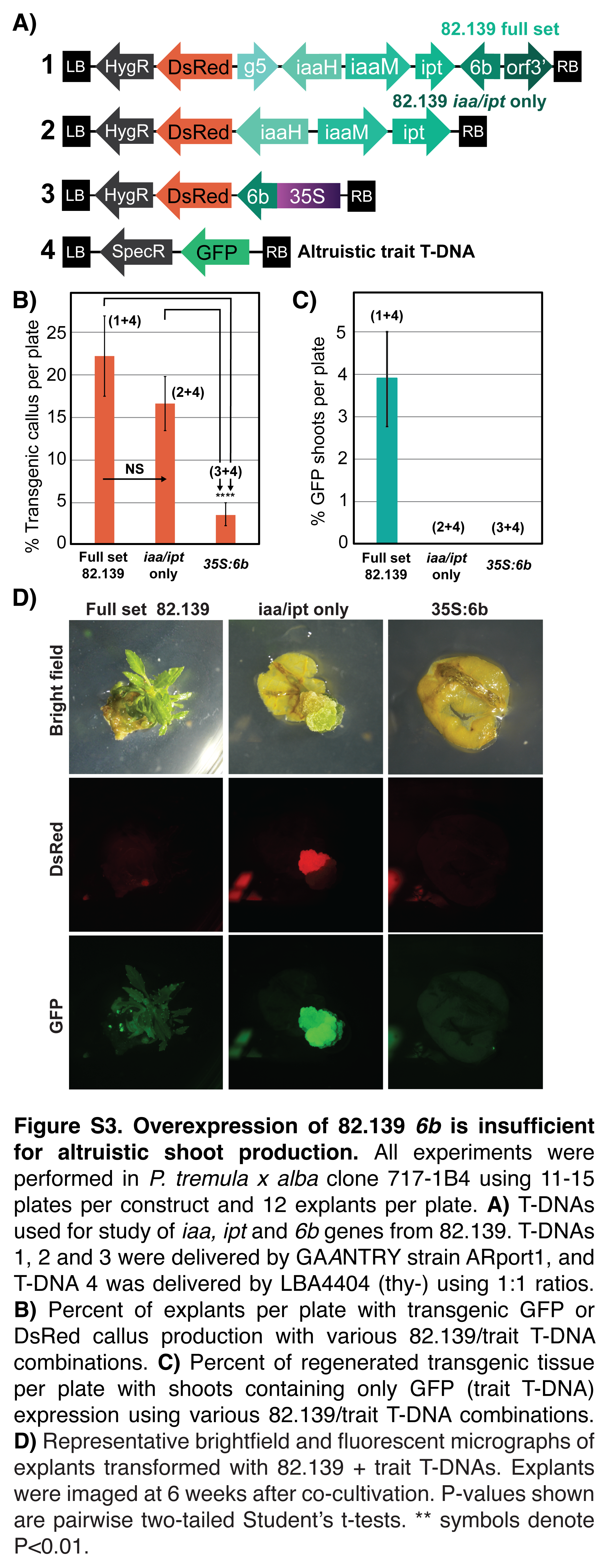

Supplement: Supplementary file 3 — Figure S3 Overexpression of 82.139 6b is insufficient for altruistic shoot production. All experiments were performed in P. tremula × alba clone 717‐1B4 using 11–15 plates per construct and 12 explants per plate. (a) T‐DNAs used for study of iaa, ipt and 6b genes from 82.139. T‐DNAs 1, 2 and 3 were delivered by GAANTRY strain ARport1, and T‐DNA 4 was delivered by LBA4404 (thy‐) using 1:1 ratios. (b) Percent of explants per plate with transgenic GFP or DsRed callus production with various 82.139/trait T‐DNA combinations. (c) Percent of regenerated transgenic tissue per plate with shoots containing only GFP (trait T‐DNA) expression using various 82.139/trait T‐DNA combinations. (D) Representative brightfield and fluorescent micrographs of explants transformed with 82.139 + trait T‐DNAs. Explants were imaged at 6 weeks after co‐cultivation. P‐values shown are pairwise two‐tailed Student's t‐tests. ** symbols denote P < 0.01. [file PBI-23-3841-s005.png]

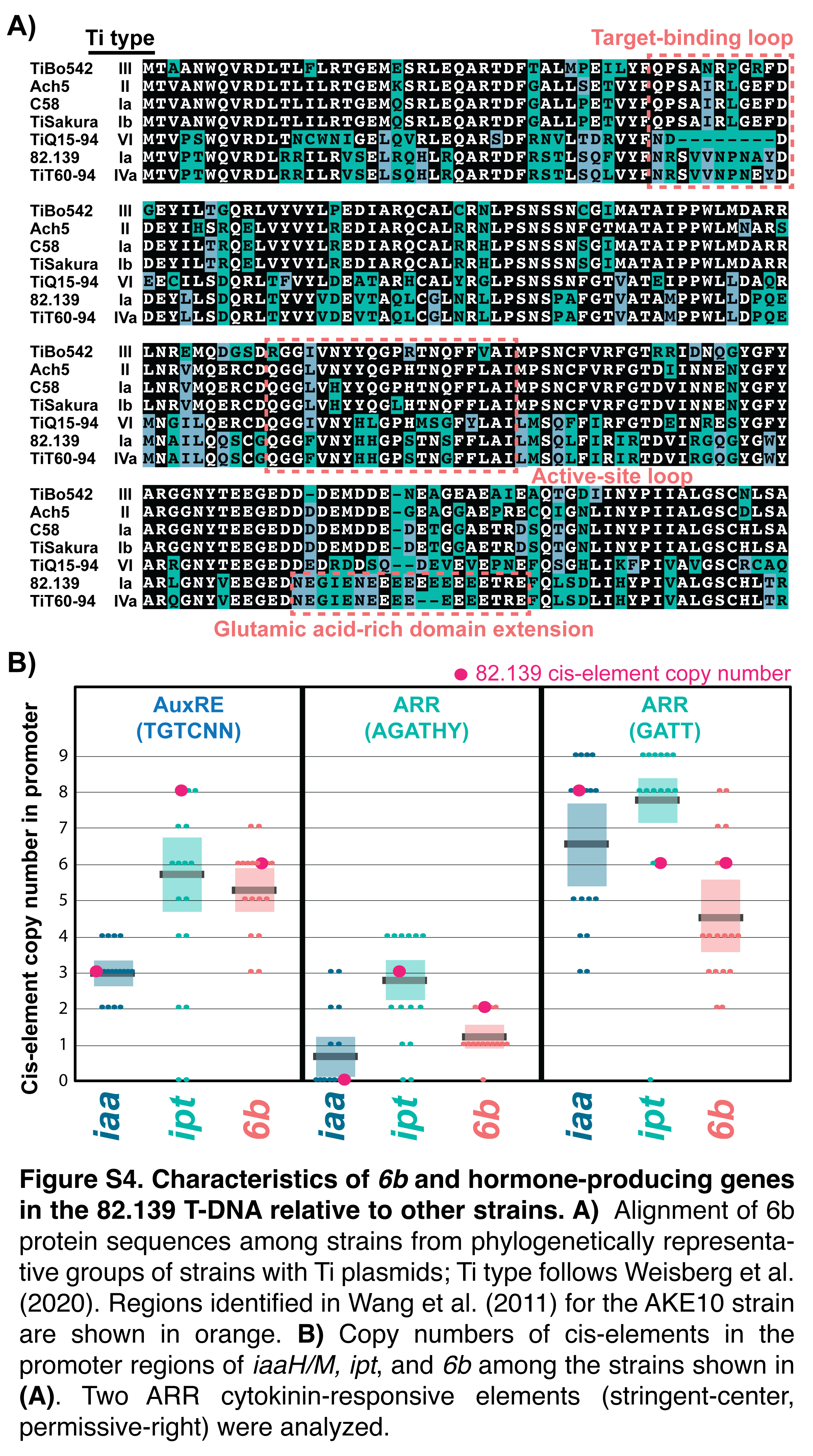

Supplement: Supplementary file 4 — Figure S4 Characteristics of 6b and hormone‐producing genes in the 82.139T‐DNA relative to other strains. (a) Alignment of 6b protein sequences among strains from phylogenetically representative groups of strains with Ti plasmids; Ti type follows Weisberg et al. (2020). Regions identified in Wang et al. (2011) for the AKE10 strain are shown in orange. (b) Copy numbers of cis‐elements in the promoter regions of iaaH/M, ipt and 6b among the strains shown in (a). Two ARR cytokinin‐responsive elements (stringent‐center, permissive‐right) were analysed. [file PBI-23-3841-s002.png]
