# Supplementary material for: Co‐transformation using T‐DNA genes from Agrobacterium strain 82.139 enhances regeneration of transgenic shoots in Populus
Source: Plant Biotechnol J. 2025 Jun 16;23(9):3841–50. doi: 10.1111/pbi.70159 (PMC12392948; doi:10.1111/pbi.70159)
Supplement: Supplementary file 7 — Table S1 Media compositions. [file PBI-23-3841-s004.pdf]

Table S1. Media compositions

Media name	Basal media	Gelling agent	Other additives	PGRs	Antibiotics
MS-induction	0.5x MS, 0.18% D(+) galactose		200mg/L L-glutamine, 250mg/L MES, 100mg/L myo-inositol, 10ml/L F-vitamin cocktail, 100mg/L acetosyringone		
CIM+thy	1xMS, 3% sucrose	3g/L Phytoblend, 2g/L Phytagel	200mg/L L-glutamine, 250mg/L MES, 100mg/L myo-inositol, 10ml/L F-vitamin, thymidine 25mg/L	1.01mg/ml 2ip, 1.86mg/L NAA	
CIM0TCR	1xMS, 3% sucrose	3g/L Phytoblend, 2g/L Phytagel	200mg/L L-glutamine, 250mg/L MES, 100mg/L myo-inositol, 10ml/L F-vitamin cocktail		200 mg/L timentin, 200mg/L cefotaxime and 30mg/L rifampicin
CIMTC+spec	1xMS, 3% sucrose	3g/L Phytoblend, 2g/L Phytagel	200mg/L L-glutamine, 250mg/L MES, 100mg/L myo-inositol, 10ml/L F-vitamin cocktail	1mg/ml 2ip, 1.86mg/L NAA	10 mg/L spectinomycin, 200 mg/L timentin, 200mg/L cefotaxime
CIM0TCR+spec	1xMS, 3% sucrose	3g/L Phytoblend, 2g/L Phytagel	200mg/L L-glutamine, 250mg/L MES, 100mg/L myo-inositol, 10ml/L F-vitamin cocktail		10 mg/L spectinomycin, 200 mg/L timentin, 200mg/L cefotaxime and 30mg/L rifampicin
2SIMTC+spec	1xMS, 3% sucrose	3g/L Phytoblend, 2g/L Phytagel	200mg/L L-glutamine, 250mg/L MES, 100mg/L myo-inositol, 10ml/L F-vitamin cocktail	0.044mg/L TDZ	200 mg/L timentin, 200mg/L cefotaxime
SB0.1TC (elongation)	1xMS, 3% sucrose	3g/L Phytoblend, 2g/L Phytagel	200mg/L L-glutamine, 250mg/L MES, 100mg/L myo-inositol, 10ml/L F-vitamin cocktail	0.225mg/L BAP	200 mg/L timentin, 200mg/L cefotaxime
Propagation	0.5x MS, 2% sucrose	7g/L agar	200mg/L L-glutamine, 250mg/L MES, 100mg/L myo-inositol, 10ml/L F-vitamin cocktail	(0.1mg/L IBA for genotype 353)	

- All media adjusted to pH 5.8, except MS-induction which is at pH 5.0
- F-Vitamin cocktail: Nicotinic Acid, 0.1g/L, Pyridoxine HCL 0.1g/L, Calcium pantothenate 0.1g/L, Thiamine HCL 0.1g/L, L-cysteine 0.1g/L, Biotin 0.5mg/L
